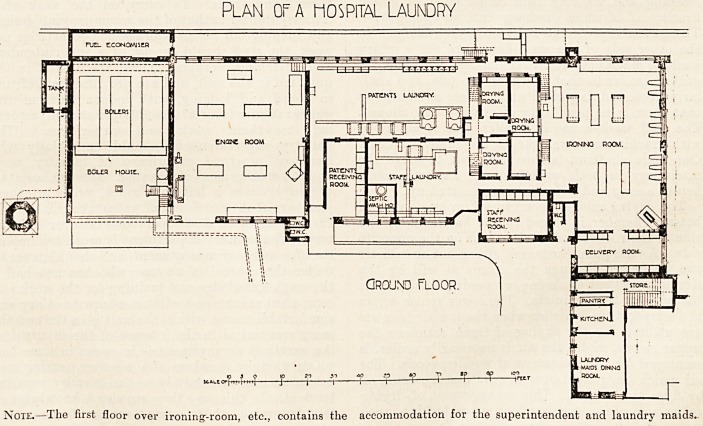# The Units of General Hospital Construction

**Published:** 1907-07-13

**Authors:** 


### HOSPITAL ADMINISTRATION.

### CONSTRUCTION AND ECONOMICS.

### THE UNITS OF GENERAL HOSPITAL CONSTRUCTION.

#### THE LAUNDRY UNIT OF A GENERAL HOSPITAL.

The various wash-houses comprised in the laundry unit should be arranged as a single-story building, and should form a complete unit in which provision is made for the housing of the staff. The extent of this unit will depend upon the size of the hospital, and the number of articles to be dealt with each week. But in all cases it should be a detached building adjoining the boiler-house and power-station, and, where possible, it should be placed on the lowest portion of the site in rear of the main buildings.

The accompanying plan shows a hospital laundry capable of dealing with 23,000 articles per week. With an additional washing machine and hydro-extractor, for which there is ample floor space, much more work could be accomplished. This laundry unit consists of two separate wash-houses, and in addition to those, which are for general cleansing, there is a special room provided for the treatment of all articles which have come from skin or septic wards, or which for any other reason may require special treatment. This room should form part of every modern hospital laundry, although the necessity for it is frequently overlooked in the design. It should have a separate entrance, and contain two large steeping tanks and a boiler. Suspicious articles are steeped in a disinfector, and are thereafter boiled previous to being passed into the general wash-house.

The general wash-houses are two in number, one for ward and patients' linen, the other for that of the staff. Adjoining these wash-houses are the drying rooms, also arranged in pairs. Opening from these is the mangling and ironing room, and as all the articles are thoroughly cleansed before reaching this stage of their treatment, this room is not sub-divided into patients' department and staff department. With regard to the buildings themselves, the interior walls of all the rooms should be lined with glazed brick, the floors of the wash-houses should be of concrete, and the floors of the ironing room of some hard wood.

In dealing with the interior fittings it may be well to follow the course of the linen from the time it leaves the ward until its return. In this way we may take a comprehensive view of the whole laundry operations. In a previous article, The Hospital, May 11) reference was made to the soiled linen bag there illustrated, which is used in each ward. The proper use of this bag obviates the repeated handling of soiled linen, as each article is noted in the laundry book at the time of its being placed in the bag, and is not again touched until it reaches the laundry. The bag is simply unhitched and conveyed to the laundry, where it is cleaned along with its contents.

Each ward is provided with a special laundry book with perforated foils for each day of the week which are detached and sent with the linen, and a counterfoil which is retained by the nurse in charge.

PLAN OF A HOSPITAL LAUNDRY

Note.—The first floor over ironing-room, etc., contains the accommodation for the superintendent and laundry maids.

This book contains a list of all the articles, and the nurse simply fills in the number of each. It is unnecessary to illustrate this in detail. The accompanying form will suffice to show the principle.

.......................................190

Ward No. Ward............... Ward...............

| Date | Mon. | Tues. | Wed. | Thurs. | Fri. | Sat. | Correct | Articles not Returned | Saturday..............190 | Friday..................190 |
|---|---|---|---|---|---|---|---|---|---|---|
| Sheets ... ... | | | | | | | | | Sheets. | Sheets. |
| Draw Sheets ... | | | | | | | | | Draw Sheets. | Draw Sheets. |
| Mattress Slips ... | | | | | | | | | Mattress Slips. | Mattress Slips. |
| Bolster Slips ... | | | | | | | | | Bolster Slips. | Bolster Slips. |
| Pillow Slips ... | | | | | | | | | Pillow Slips. | Pillow Slips. |
| Grey Pillow Slips, etc., etc. | | | | | | | | | Grey Pillow Slips, etc. | Grey Pillow Slips, etc. |

The counterfoil includes all the days of the week, and a column for returns.

### The Laundry.

At the entrance to the patients' wash-house is a receiving room for the soiled linen, and this is fitted with open bins for the classification of the different articles. At the entrance to the staff wash-house a similar receiving room is provided. The wash-house for patients' clothing is fitted on one side with steeping and washing tubs constructed of glazed fireclay, all of which discharge into an open gutter. This gutter is protected at its end by a grating. On the other side of the wash-house are three rotary washing machines and two centrifugal wringers. A gutter similar to that on the other side, and similarly protected, is placed under these machines, so that there is no possibility of drains being choked. The staff wash-house is smaller, but is equipped with machine and wringer on the same principle. One main shafting is thus sufficient to drive the machinery in both wash-houses. In both wash-houses a soap and soda dissolver is fitted up.

The drying rooms are arranged on two flats. A hot-air chamber is placed in the basement at one side of the rooms, and electric fans on the opposite side. As the plan illustrates, either hot or cold air can be drawn through the rooms at will by the simple expedient of closing or opening the dampers placed over the air inlets. The lower rooms where the temperature is higher when the hot air is in use are utilised for the drying of linen articles; the upper rooms, where the air is necessarily cooler, is employed for the treatment of woollen goods. The air which has passed over the clothes is carried off by flues in the roof. This arrangement of drying rooms not only economises floor space, but is a much more efficient method than outdoor drying, which in a large town can never be satisfactory. Atmospheric conditions, smoke, and uncertain weather render the outdoor method uncleanly and unreliable where thousands of articles are dealt with and the time and labour involved in conveying to a bleaching green, where such exists, adds materially to the wages bill. Clothes dried by this method where there is a constant current of fresh air are dried much more expeditiously, and are left clean and sweet. The old method of using clothes-horses in a chamber of hot but still air was not only a more tedious process, but had the disadvantage of giving the clothes a musty smell.

The linen which has passed through the washing and wringing machines and the drying rooms is then passed into the mangling and ironing room. The variety of mangles, ironers, collar and cuff machines, goffering machines, gas and electric irons, also the variety of washing and wringing machines, are so numerous that it is not our present intention to particularise any pattern of either. In a modern hospital laundry the bulk of the work is done by machinery, although some laundresses prefer hand irons for special work, such as the dressing of shirts: provision must therefore be made for the heating of these hand irons. To carry on the work of a laundry similar to that of the accompanying design, and turning out on an average 23,000 articles per week, the staff should consist of a superintendent and 18 female workers. These are housed in the unit, fed in the unit, and are provided, in addition to board and lodging, with indoor uniform and wages ranging from £10 to £20 per annum, according to their work and experience. The laundry superintendent should be a lady who has had practical experience of laundry work and the management of servants. In some hospitals the supervision is left to a nurse, while in others members of the nursing staff undertake this duty in turn. This method of supervision is never satisfactory. The laundry department is much too important for amateur management, and should never be under the control of anyone who has not had a thorough knowledge and training for the work. A competent manageress with an adequate salary and comfortable rooms within the unit is in the end the more economical in the interest of the institution. An excellent arrangement is in vogue in some hospitals whereby members of the senior nursing staff act in turn as understudy to the laundry superintendent. In this way they acquire a knowledge of laundry work under competent guidance which proves invaluable to them should they become matrons of small hospitals.

Reference has been made to an average of 23,000 articles per week. This works out roughly as 16,000 articles for ward and patients' use: and between 6,000 and 7,000 for nurses and officials. The cost of materials, such as soap, soda, etc., for this quantity averages £2 14s. 6d. per week. The cost of water varies so much in different districts that no average can be taken. In some cases the local authorities allow a certain quantity free of charge to charitable institutions.

## Figures and Tables

**Figure f1:**